# News and Coming Events

**Published:** 1909-08-28

**Authors:** 


### News and Coming Events.

The returns of the Registrar-General show a decline of 1.9 per 1,000 in the birth-rate in the quarter ending June 30, as compared with the average of the same period over the preceding ten years. The death-rate has similarly declined by 1.3 per 1,000.

A United Charities Fund has been created in Sydney to meet the objection raised by the City Council against separate street collections on behalf of individual institutions. These have now combined to collect for the fund in much the same way as the Hospital Saturday Fund, and 1,700 collectors had been engaged in September 1908, to work 107 districts, including the city and suburbs.

The Metropolitan Ambulances Bill was discussed last week before the Standing Committee A of the House of Commons, and finally ordered to be reported. The Bill, which is presented by Sir W. Collins and influentially backed virtually reproduces the powers sought by the London County Council three years ago, in order to enable them to provide for London such ambulance service as the Departmental Committee's investigations have shown to be required. Then the Council sought powers to establish and maintain an efficient ambulance service for cases of street accident and illness occurring in London. The clauses passed the House of Commons, but did not pass the Lords. The Home Office thereupon appointed a Departmental Committee, which reported that it had been abundantly shown that the present system of dealing with cases of accident and sudden illness occurring in streets and public places within the metropolis was gravely defective, and resulted in much preventable detriment and suffering. The Departmental Committee was divided in opinion as to whether the London County Council or the Metropolitan Asylums Board should be the authority for ambulance purposes in London. In the course of the discussion on the present Bill, Mr. Gladstone, speaking in its support, said the Metropolitan Asylums Board did very excellent work, but to invest it with the whole ambulance work of London would be to give it duties and an authority for which it was not constituted, and, on the whole, for which it was not specially designed. There was not the smallest chance of getting the House of Commons to accept the Asylums Board as the authority, and, if so, to press forward such a proposal would cause dangerous delay. This was a matter of urgency. Something was required to be done, and sooner than shelve the matter indefinitely he hoped this Bill would be accepted. Sir W. Collins mentioned that the whole origin of the County Council's move in this matter was the offer in 1902, or thereabouts, by a well-known philanthropist to present the County Council with an up-to-date automobile ambulance, and the County Council found that they had no power to spend money in maintaining such a service. The cost of maintaining a self-contained station like that at St. Bartholomew's was £1,500 a year, and it was estimated that ten stations would suffice for the County of London.

Mr. Sydney Scott, M.S., F.R.C.S., has been appointed Surgeon for Diseases of the Throat and Ear at the National Hospital for the Paralysed and Epileptic, Queen Square. Mr. Scott, who is Assistant Aural Surgeon at St. Bartholomew's Hospital, was formerly Chief Assistant in the Aural Department at St. Bartholomew's Hospital, and Aural Surgeon to the Evelina Hospital for Children.

An exhibition of memorials of Darwin was opened on the 16th inst. at the Natural History Museum, South Kensington, and will remain open for some time. The exhibition commemorates the centenary of Darwin's birth and the fiftieth anniversary of the publication of his principal work. The guide-book is written by Dr. W. G. Ridewood, and practically gives the story of Darwin's life. The main object of the exhibition is to illustrate the chief arguments of " The Origin of Species," and much of the material which actually passed through Darwin's hands and the various species which he had in view when he wrote are for the first time brought together for the inspection of the public. Some of the manuscripts shown are particularly interesting.

The new male hospital pavilion erected in the grounds of Tynemouth Union Workhouse was formally opened in July last. The hospital is situated at the north-west corner of the workhouse premises, and faces Preston Road. It is 184 ft. long, and consists of two floors. There are on the ground floor two wards, the larger one 84 ft. by 24 ft., and the smaller one 48 ft. by 24 ft., with accommodation for twenty-eight beds and sixteen beds respectively, the necessary sanitary arrangements being provided in offshoots in the centre of each ward. Separating the two wards is a day room, 42 ft. by 16 ft., with large bay windows on either side; two separation wards, each containing two beds, are also provided, together with service room and bathroom. The upper floor is a replica of the ground floor, with the addition of a glass-roofed balcony, with access thereto from the large ward, for the open-air treatment of patients. The total accommodation thus provided is for ninety-six beds. At each end of the building panic staircases have been placed. Large airing courts surround the building, and the sanitary conditions are on the most up-to-date principles. Special attention has been paid to the heating of the building, which is carried out on the hot-water system, by means of radiators, heated by a calorifier in the basement, while in the case of emergency Shorland's "Manchester" stoves, with underground flues, have also been installed. The approximate cost has amounted to £5,900, which works out at about £61 per bed. The building has been erected from the plans and specifications of Mr. Henry Gibson, architect, the contractors being Mr. Jos. F. Newbold, both of North Shields. The sub-contractors were:—Mr. H. Taylor, iron and plumbing work; Messrs. W. W. Ward and Sons, painting and decorating; and Messrs. Dinning and Cooke, hot-water engineers.

The Lord Provost, magistrates, and town council of the city of Glasgow have granted a site in the West-end (Kelvingrove) Park for the Scottish Exhibition of National History, Art, and Industry, to be held there in 1911. They have also agreed to support the undertaking and to do everything possible to render it a success.

We are requested to state that copies of the Revised Syllabus of Physical Exercises, issued by the Board of Education for use in elementary schools and in training colleges, to which we refer in an annotation in the present issue, will not be available for public distribution until the third week in September.

It is announced that the King has been pleased to give effect to the representations of the General Council of Medical Education and Registration that it is expedient to confer on the registered practitioners resident in England and Wales the power of returning an additional member to the General Council, but that the addition in question should not be made until the next ensuing general election of direct representatives.

It was recently announced that the Orient Line had granted free first-class tickets between Australia and England to selected scholars of the Australian Universities, and the three first nominations have now been made by the Chancellor of the Sydney University: Messrs. H. J. Swain, Science Research Scholar; S. G. Lusby, Barker Mathematical Scholar; and H. K. Archdale, Woolley Classical and Philosophical Scholar. The three gentlemen selected will travel to England by the Orient Line R.M.S. *Omrah.*

In a letter to the *Times,* Messrs. Lucas and Murray, of the Hospital for Sick Children, Great Ormond Street, state that the net profits of the Midsummer Fair and Fête on behalf of the hospital amount in round numbers to £2,610, and the donations received for this special occasion to £2,777, making a total sum of £5,387 handed over to the hospital. After receiving this sum the hospital still owes £5,500 to its bankers for money advanced to carry on the necessary work for the current year.

An important step has been taken by the Local Government Board in the direction of reducing infantile blindness. Orders have just been issued extending the application of the Infectious Disease (Notification) Act, 1889, to the disease known as *ophthalmia neonatorum* in the five pottery towns of Burslem, Stoke-on-Trent, Longton, Newcastle-under-Lyme, and Fenton. The effect of the orders is that this disease is added to the list of diseases compulsorily notifiable under the provisions of the Act. This step has been taken on the application of the local authorities concerned, and the Board is understood to be willing to entertain similar applications from other districts.

After having been the subject of an action in the Probate Division of the High Court, in which action the President, on July 7, pronounced in favour of its validity, the will has now been proved of Mr. Hugh Lewis, of Woodfield House Hill, Sutton Coldfield, Warwick, formerly in business as a pawnbroker at Tipton, Staffs., who died on August 21, 1908, at the age of 76 years. His property is valued for probate at £90,572 10s. gross, of which the net personalty has been sworn at £84,240 2s. 1d. The will is made on a sheet of notepaper, and the testator bequeaths all his property to the Guest Hospital, Dudley. The estate duty on the property will amount to about £7,000, leaving a total gross amount for the hospital of over £80,000. This will have to pay about £8,000 legacy duty, which will reduce the amount available for the hospital to a net sum of between £70,000 and £75,000.

The death occurred on Wednesday last week of Mr. Sydney George Lushington. Mr. Lushington was a member of the Northern Circuit, and standing counsel to the General Medical Council.

The eleventh annual conference of the American Hospital Association, now composed of about 700 of the leading hospital officials of the United States and Canada, will be held at Washington, D.C., September 21-24.

Professor Guignard, Director of the Paris School of Pharmacy, read a report at the last meeting of the Academy of Medicine on researches made by MM. Perrot and Goris concerning the sterilisation of fresh medicinal plants by a scientific method and their uses for making galenicals. The method of sterilisation is based on the destruction of the ferments, and the plants thus treated retain the colour and taste of fresh plants. The dried plants become a raw material which can be used for preparing galenicals at any time of the year. MM. Perrot and Goris claim that they have obtained physiological vegetable extracts comparable in therapeutical action with the fresh plant.

At the City Coroner's Court, Dr. F. J. Waldo inquired as to the death of Annie Bird, aged 25, wife of William Henry Bird, of Liverpool Road, Islington, who died in St. Bartholomew's Hospital on Wednesday in consequence of burns received on Monday. Her clothing caught fire owing to a defective gas ring, and a verdict of accidental death was returned. The coroner, referring to the need of motor ambulances, said that upwards of 14,000 street accidents occurred last year in London alone. The amount of unnecessary suffering must be tremendous, and he had been told by doctors that many deaths would have been prevented if the patients could have been got to hospital with greater celerity.

The twenty-third annual report of the Holloway Sanatorium at Virginia Water states that last year the daily average of patients and voluntary boarders was 376. The number of cases admitted at reduced rates was 191, of which 27 were received free of charge, a sum of £9,555 being expended in charity in the maintenance of patients at a cost below the actual maintenance rate. The new male infirmary, which cost £6,700, is now in occupation, and has proved a most valuable addition. The total expenditure for 1908 was £59,434 and the income £67,248, leaving a credit balance of £7,813. The average weekly expenditure per patient was £2 11s. The report of the medical superintendent states that the recovery rate was 37.27 per cent., and the death rate 6.84, whilst the average age at death was sixty-two.

### THE PURE FOOD CONGRESS.

The arrangements for the forthcoming Pure Food Congress, which will be held in Paris from October 17 to 24, are rapidly approaching completion. The general correspondent for the United Kingdom is Mr. Loudon M. Douglas, of 3 Lauder Road, Edinburgh. Among the delegates who have been officially appointed are the Hon. J. W. Taverner, Agent-General for Victoria, and the Agents-General for Tasmania and New South Wales; Professor W. R. Smith, representing the Royal Institute of Public Health; Dr. R. R. Tatlock, the Society of Public Analysts; Mr. Richard Robinson, the Department of Sanitary Control in the Borough of Westminster; and Mr. Alfred C. Chapman, Secretary of the Society of Public Analysts of the United Kingdom. In addition to these delegates there will be many others representing scientific societies, medical associations, and firms and companies interested in the various branches of the food industries.

